# Climate Drives the Meningitis Epidemics Onset in West Africa

**DOI:** 10.1371/journal.pmed.0020006

**Published:** 2005-01-25

**Authors:** Benjamin Sultan, Karima Labadi, Jean-François Guégan, Serge Janicot

**Affiliations:** **1**IRD–Laboratoire d'Océanographie Dynamique et du Climat (LODYC)–UMR 7617 (CNRS/IRD/P6/MNHN)–Université Pierre et Marie CurieParisFrance; **2**Université de Paris 7 Denis Diderot UFR GHSS (c.c. 7001) Dynamique des Milieux et Risques–2ParisFrance; **3**IRD–GEMI-UMR 2724 IRD-CNRS, Evolution des Systèmes SymbiotiquesMontpellierFrance; Australian National UniversityAustralia

## Abstract

**Background:**

Every year West African countries within the Sahelo-Sudanian band are afflicted with major meningococcal meningitis (MCM) disease outbreaks, which affect up to 200,000 people, mainly young children, in one of the world's poorest regions. The timing of the epidemic year, which starts in February and ends in late May, and the spatial distribution of disease cases throughout the “Meningitis Belt” strongly indicate a close linkage between the life cycle of the causative agent of MCM and climate variability. However, mechanisms responsible for the observed patterns are still not clearly identified.

**Methods and Findings:**

By comparing the information on cases and deaths of MCM from World Health Organization weekly reports with atmospheric datasets, we quantified the relationship between the seasonal occurrence of MCM in Mali, a West African country, and large-scale atmospheric circulation. Regional atmospheric indexes based on surface wind speed show a clear link between population dynamics of the disease and climate: the onset of epidemics and the winter maximum defined by the atmospheric index share the same mean week (sixth week of the year; standard deviation, 2 wk) and are highly correlated.

**Conclusions:**

This study is the first that provides a clear, quantitative demonstration of the connections that exist between MCM epidemics and regional climate variability in Africa. Moreover, this statistically robust explanation of the MCM dynamics enables the development of an Early Warning Index for meningitis epidemic onset in West Africa. The development of such an index will undoubtedly help nationwide and international public health institutions and policy makers to better control MCM disease within the so-called westward–eastward pan-African Meningitis Belt.

## Abstract 2

An analysis of winds and yearly meningitis epidemics in West Africa shows a surprisingly close correlation that could help to predict disease outbreaks

## Introduction

Meningococcal meningitis (MCM) has affected Sahelian Africa for centuries and became endemic over the past 25 y. During the 1980s, the World Health Organization (WHO) registered between 25,000 and 200,000 disease cases per year, with about 10% of them resulting in death, and with the highest infection rates observed in younger children [1]. MCM became, therefore, a public health concern in the poorest regions in the world following the severe drought at the end of the 1970s.

MCM is an infection of the meninges, caused by the bacteria Neisseria meningitidis, that causes high death rates in African communities. The agent is highly contagious, and person-to-person aerial transmission occurs through respiratory and throat secretions [2]. Interaction between different environmental parameters (e.g., immune receptivity of individuals, a poor socioeconomic level, the transmission of a more virulent serotype [such as the recent emergence of the serogroup W135 in West Africa], social interactions [such as pilgrimages, tribe migrations, and meetings], and some specific climatic conditions) may play a part in MCM disease outbreaks and spread within local populations [2].

**Figure 1:**
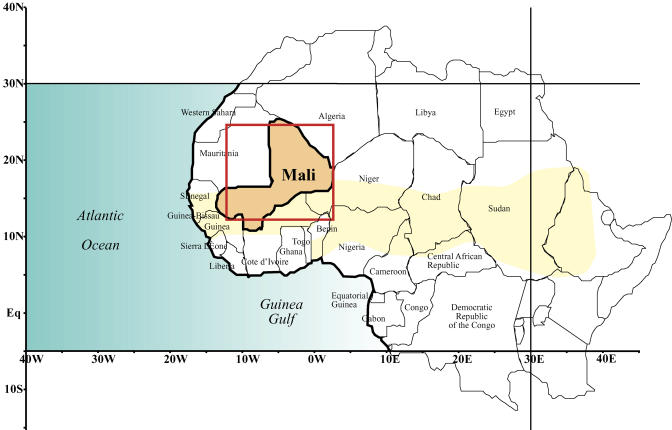
The “Meningitis Belt” in West Africa Modified from the WHO [9].

Among favorable conditions for the resurgence and then dispersion of the disease, climatic conditions may be important as environmental forces inducing periodic fluctuations of disease incidence. Recent findings concerning the population dynamics of some infectious diseases have clearly identified the importance of climate as a major driver [3,4]. MCM outbreaks in West Africa usually start at the beginning of February, and then disappear in late May. The geographical distribution of disease cases is called the “Meningitis Belt” and is roughly circumscribed to the biogeographical Sahelo-Sudanian band [5,6]. This Sahelo-Sudanian region has a dry winter, dominated by northern winds, called the Harmattan, followed by a wet season starting in spring with the monsoon. The co-occurrence in both space and time of MCM disease cases and climate variability within the Sahelo-Sudanian area suggests that the occurrence of MCM might be directly related to climate. So far, very few studies have tried to quantify the potential linkages that could exist between climate and MCM outbreaks (Figure 1).

The winter climate causes damage to the mucous membranes of the oral cavity through dry air and strong dust winds, and creates propitious conditions for the transmission of the bacteria responsible for MCM; low absolute humidity and dust may enhance meningococcal invasion by damaging the mucosal barrier directly or by inhibiting mucosal immune defenses. In contrast, higher humidity during both the spring and summer seasons strongly reduces disease risk by decreasing the transmission capacity of the bacteria [7,8], and MCM epidemics generally stop with the onset of rainfall [9]. In addition to the seasonal cycle, the link between climate and meningitis has also been documented at the interannual scale in northern Benin, where Besancenot et al. [10] have suggested a positive relationship between low absolute humidity and interannual variability in meningitis. Meanwhile, although the global influence of climate is quite clear, the effects of climatic variability on MCM population dynamics are still only partially known because of the mixing of different processes acting at different spatial hierarchical scales, and the interactions between disease outbreaks and medical, demographical, and socioeconomic conditions.

Most studies thus far have focused on very small spatial scales, and the need remains to discriminate between local properties and potential large-scale effects in disease patterns, to go beyond data heterogeneities and idiosyncratic details in order to identify important disease patterns influenced by large-scale forces such as climate variability (see Methods). The aim of the present work is thus to document the climatic context of MCM disease outbreaks and population dynamics in a highly affected Sahelian country, that is, Mali, and to show, if it exists, the presence of a correlation between climate and seasonal resurgence of disease. The idea behind the present study was to explain MCM disease dynamics in Mali in a statistically robust way, which will permit us to propose some tools for predicting disease risks for the benefit of public health.

## Methods

### The Scaling-Up Approach: From Local to Global Scales

Recent findings concerning the population dynamics of some infectious diseases have clearly identified the importance of climate as a major driver [3,4]. With evidence of the impact of large-scale meterological phenomena such as El Niño on infectious disease patterns, modern epidemiology is now confronted with a scale problem in the identification of the spatiotemporal scales that might be relevant to explain patterns and processes [11]. Since most previous studies have focused on very small spatial scales, there is a need for “bottom-up” approaches to discriminate between local properties and potential large-scale effects on disease patterns. One of the simplest assumptions of these “bottom-up” approaches is the assumption that local scales are random processes overlaying a driving large-scale phenomenon such as climate variability. As such, this scaling-up approach seeks to point out the emerging patterns conditioned by the large-scale processes, with a random or deterministic function *f* such that:


Local data = *f*(large-scale forces, idiosyncratic details).

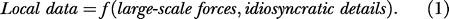



The aggregation of local data in the scaling-up approach is a simple way to go beyond data heterogeneities and idiosyncratic details so that only the important disease effects of large-scale forces remain. That is the rationale for our study: to show that large-scale phenomena such as the seasonal Harmattan winds over the whole of Mali can contribute to the periodic resurgence of MCM and its variation in time on a national scale, even if this aggregate analysis for the entire country is not able to capture variations at smaller space scales.

### Epidemiological Data: The WHO Weekly Reports

The diagnosis of MCM is based on both physical examination and on evaluation of the cerebrospinal fluid (CSF) from a lumbar puncture. As the disease is characterized by a sudden onset of intense headache, fever, nausea, vomiting, photophobia, and stiff neck, in association with neurological symptoms (lethargy, delirium, coma, and convulsions), the WHO [9] recommends that the clinical diagnosis include an examination for meningeal rigidity, neurological signs, purpura, blood pressure, and focal infection. A lumbar puncture and CSF examination are then used to confirm the diagnosis based on physical examination and to identify the meningococcus [9]. This diagnosis is the basis for disease surveillance and case reporting using a standard case definition for MCM (Box 1) that can be implemented in any health-care setting. Meningitis reports are included in the weekly reports of notifiable diseases and are aggregated at different spatial scales from the health site to the country level. The present study is based on these weekly reports by the WHO's Department of Communicable Disease Surveillance and Response of cases and deaths due to MCM over the whole of Mali from 1994 to 2002.

**Box 1. Standard Case Definition of MCM Modified from the WHO [9]**

This case definition allows the detection of cases of meningococcal septicemia.

1. **Suspected case of acute meningitis^a^.** Sudden onset of fever (>38.5 °C rectal or 38.0 °C axillary) with stiff neck. In patients under 1 y of age, a suspected case of meningitis occurs when fever is accompanied by a bulging fontanelle.
2. **Probable case of bacterial meningitis^b^.** Suspected case of acute meningitis as defined above with turbid CSF.
3. **Probable case of MCM^b^.** Suspected case of either acute or bacterial meningitis as defined above with Gram stain showing Gram-negative diplococcus or ongoing epidemic or petechial or purpural rash.
4. **Confirmed case^c^.** Suspected or probable case as defined above with either positive CSF antigen detection for N. meningitides or positive culture of CSF or blood with identification of *N. meningitides.*

^a^Often the only diagnosis that can be made in dispensaries (peripheral level of health care).

^b^Diagnosed in health centers where lumbar punctures and CSF examination are feasible (intermediate level).

^c^Diagnosed in well-equipped hospitals (provincial or central level).

### The Typical Seasonal Pattern of the Disease

In this study, an epidemic is defined in terms of population dynamics following Anderson and May [12] and Grenfell and Dobson [13]. This definition considers disease resurgence and its variation in time and allows us to focus on the cyclic character of the disease resurgence each year, even if the number of annual cases is low, and it makes it easier to find temporal correlations between climate and disease. To represent a typical seasonal cycle of a meningitis epidemic, we computed the weekly mean of standardized anomalies *M*(*w*) of the number of cases as



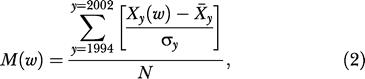



where X̄_y_ and *σ_y_* represent, respectively, the mean and the standard deviation of the 54 weekly values of cases *X_y_*(*w*) for the year *y,* and *N* = 9 represents the number of years for the 1994–2002 period.

### The Onset of the Epidemic

We determined the week of the onset of the epidemic for each year by characterizing a breaking slope in the annual cycle of the number of cases. The dates of the breaking slope have been determined objectively by using the Mann–Whitney–Pettitt test [14], which is a nonparametric test used here to detect a “change point” in a time series. A change point is defined as a point on either side of which values are on average higher or lower than the whole of the other data points. Considering the studied time series *X_y_*(*w*), we computed *U_y_*(*w*) as



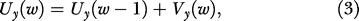



where 2 ≤ *w* ≤ 54 and *U_y_*(1) = *V_y_*(1), where *V_y_*(*w*) is defined by



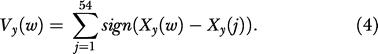



Then the the most significant change point of the year *y* is the point for which the value*|U_y_*(*w*)| is maximized. The probability *P_y_*(*w*) of a given week being a change point is defined by



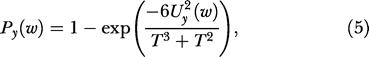



where *T* = 54 (the length of the time series in weeks).

### Atmospheric Data: The NCEP/NCAR Reanalysis

The National Centers for Environmental Prediction (NCEP) and the National Center for Atmospheric Research (NCAR) have completed a reanalysis project with a current version of the Medium Range Forecast model [15]. This dataset consists of a reanalysis of the global observational network of meteorological variables (wind, temperature, geopotential height [i.e., the height of a pressure surface above mean sea level], humidity on pressure levels, surface variables, and flux variables such as precipitation rate), with a “frozen” state-of-the-art analysis and forecast system at a triangular spectral truncation of T62, performing data assimilation throughout the period from 1948 to the present. This analysis enables circumvention of problems involving previous numerical weather prediction analyses due to changes in techniques, models, and data assimilation. Data are reported on a 2.5° × 2.5° grid every 6 h (00.00, 06.00, 12.00, and 18.00 UTC) on 17 pressure levels from 1,000 hPa to 10 hPa, a good resolution for studying synoptic weather systems. For this study we used the wind speed fields at 1,000 hPa (near the surface) for the 9 y of the 1994–2002 period: first we averaged the four outputs of each day, and then we averaged these daily means for each week to obtain a weekly value.

### Computation of the Harmattan Wind Index

The principal component analysis (PCA) [16] is a multivariate procedure that extracts the common variance that exists in a set of variables. The main use of PCA is to reduce the size of a dataset while retaining as much information as possible in principal components (PCs), which are linear combinations of the initial variables. In this study, this technique has been used in order to summarize the spatiotemporal variability of wind fields at low levels. As the input data are spatial objects (grid points), the PCA gives for each mode a spatial pattern associated with a time series (the PC). We performed the PCA on the weekly values from 1994 to 2002 of wind speed at 1,000 hPa over the Mali window (in red in Figure 1) by taking into account all the grid points from 12.5° N to 25° N and from 12.5° W to 2.5° E. The input matrix was thus composed of 6 × 7 = 42 loadings (the number of grid points over the spatial window) and 486 scores (the number of weeks in the 1994–2002 period). Data was first standardized in order to extract the correlation matrix C = X′X, where X represents the input matrix and X′ its transpose. The α^th^ PC time series *ψ_α_* can thus be obtained by a linear combination of the initial variables through



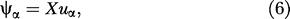



where *u*_α_ is the *α* ^th^ eigenvector of the correlation matrix C associated with the eigenvalue *λ*_α_.

The *α* ^th^ spatial pattern is the correlation map between the initial wind fields and the *α* ^th^ PC time series. The examination of the different spatial patterns and PC time series (not shown here but previously discussed in [17]) reveals a close relationship between Harmattan wind dynamics and the third PC with negative values, which represents a strong wind in southern Mali. The Harmattan wind index of the study thus represents the third PC, with a temporal pattern very similar to the seasonal cycle of wind speed associated with the Harmattan winds over Mali.

## Results

### The “Epidemic Seasonality” in Mali

**Figure 2:**
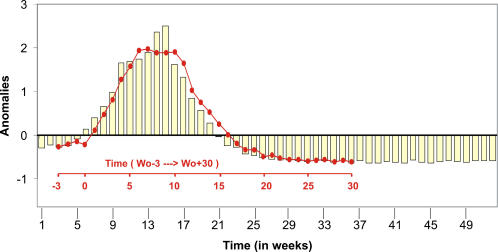
The Seasonal Periodicity of Meningitis Cases Mean seasonal pattern of the number of cases of MCM over the 1994–2002 period in standardized anomalies (bars). The red curve represents the same evolution, but in composite mean, using the week of epidemic onset as the reference date, *W*_o_, each year. Time series in red is shown from “*W*_o_ − 3 wk” to “*W*_o_ + 30 wk.”

The weekly records of the WHO's Department of Communicable Disease Surveillance and Response of cases and deaths due to MCM for the 1994–2002 period allowed us to describe the seasonal evolution of MCM epidemics in Mali. Two important parameters were used: the date of the onset of the epidemic and the date of the seasonal maximum number of cases. We determined for each year the week of the onset of the epidemic (here called “*W*_o_”) as determined by a breaking slope in the annual cycle of the number of cases. The dates of breaking slope have been determined objectively by using the Mann–Whitney–Pettitt test [14], which is a nonparametric test used here to detect a change point in a time series. This test has the advantage of being adapted to small samples, giving the point of the most significant change and the probability of it being a significant change point (see Materials and Methods). The mean date of epidemic onset fell between the fifth and sixth week of the year (7–15 February), with a standard deviation of about 2 wk (5.2 ± 1.7 wk). For the 1994–2002 period, the maximum number of cases occurred between week 13 and week 14, that is, between 1 April and 15 April, with a standard deviation of about 2 wk (13.7 ± 1.6 wk) (Figure 2).

In order to mitigate the effect of strong variability from one year to another during the 1994–2002 period, we computed the average of standardized anomalies of the number of cases (bars in Figure 2) to represent a typical seasonal pattern of a meningitis epidemic. The first 5 wk are characterized by negative anomalies. The average length of the “epidemic year,” as defined by the number of consecutive weeks with positive anomalies, is 4 mo (16 wk).

To improve the description of the seasonal pattern of the epidemic and to reduce noise due to the variability of the onset date year to year, we determined the composite mean of the number of cases over the 1994–2002 period by using the week of epidemic onset for each year as the respective reference date, *W*_o_. The red curve of Figure 2 shows the mean seasonal course before and after the onset of the epidemic, showing an abrupt increase of the number of cases—the “upward phase”—until the sixth week after the onset, a highly active period of the disease—the “active phase”—from “*W*_o_ + 6” to “*W*_o_ + 10,” followed by a decrease of the number of cases—the “downward phase”—until the end of the epidemic around 16 wk after the onset. Both upward and downward phases lasted on average 1.5 mo.

### The Atmospheric Circulation during an Epidemic in Mali

**Figure 3:**
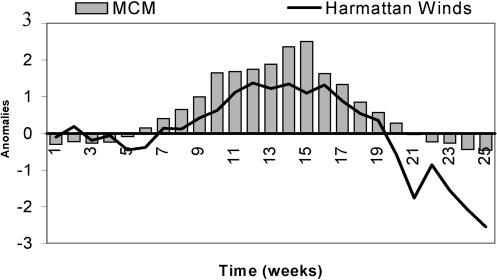
Temporal Patterns of Epidemics and Climate Weekly means of the Harmattan wind index over the 1994–2002 period and mean seasonal pattern of the number of cases of MCM (in standardized anomalies).

Rainfall distribution over West Africa is controlled by the meridional migration of the intertropical convergence zone following the seasonal excursion of the sun [18]. The latitudinal shift of this zone of high humidity and instability leads to an opposition of two main annual regimes: the bimodal regime of the Guinean latitudes (from the equator to 7° N) with two rainy seasons during spring and autumn, and the unimodal regime of monsoon over Sudano-Sahelian Africa and succession of a dry winter and a wet summer [19]. North of the intertropical convergence zone, the intertropical front is defined as the confluence line between moist southwesterly monsoon winds and dry northeasterly Harmattan [20,21]. The seasonal progression of this system, involving a migration toward the summer pole of moisture and winds converging in the low layers, can be documented by using weekly fields surface wind speed from NCEP and NCAR data, which provide gridded atmospheric parameters with a 2.5° resolution [15]. The relationship between atmospheric circulation and the seasonal course of the MCM epidemic in Mali was studied using a regional index summarizing the spatiotemporal evolution of the low-layer circulation. This index was obtained from a dominant mode of a PCA (see Materials and Methods) applied to weekly fields of surface wind speed over the 1994–2002 period in Mali. The seasonal pattern shows the Harmattan wind dynamics (Figure 3) with negative values representing strong winds, and positive values representing weak winds, in the southern part of Mali, the area under study in the present work.

**Figure 4:**
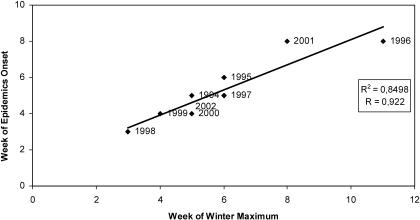
The Onset of Epidemics and the Winter Maximum Scatter plot of the week of epidemic onset and the week of winter maximum over the 1994–2002 period.

Using this atmospheric index, we defined the date of “winter maximum” as the first minimum of the wind index for each year of the period 1994–2002. The winter maximum thus corresponds to the week where wind speed is the strongest. The mean date of winter maximum is around the sixth week, with a standard deviation of 2 wk, corresponding to the week when the Intertropical Front is located at its southern latitude. The Harmattan wind index shows a temporal pattern very similar to the that of the number of cases of MCM, with a clear breaking slope at the sixth week, on 15 February, corresponding to the onset of the epidemic and to the winter maximum, and with a recession of the disease at the 16th week concomitant with the onset of the wet season in the south part of Mali in early May. It is interesting to note that although they are determined from two different datasets, the mean weeks of winter maximum and of the onset of the epidemic are identical, 7–15 February. This coherence is reinforced by a very strong correlation between the two dates (0.92) for the years 1994 to 2002. Figure 4 shows the linear regression analysis between week of winter maximum and week of epidemic onset. Although the number of years under consideration is low, the scatter plot points out the close statistical linkage between these two events, suggesting that the winter maximum explains more than 85% of the variance in the week of epidemic onset in Mali: An earlier winter is associated with an earlier onset of the epidemic, and a later winter with a later onset. However, even though the results of the correlation analysis are strongly significant, the high *R* ^2^ is partially due to the low number of considered years (only nine); this low number is the main limitation of the present analysis.

## Discussion

In this paper and a previous publication of ours [17], by using the weekly number of cases of MCM disease in Mali and large-scale fields of surface wind speed, we clearly identify a strong relation between climate and the seasonal pattern of MCM cases in Mali. It is shown that the onset of disease outbreak is characterized by a clear breaking slope in the seasonal cycle of the number of cases at the sixth week of the year, that is, 15 February. The computation of an atmospheric index based on surface wind speed over Mali points out that this abrupt shift is also present in the atmospheric signal, corresponding to the winter wind maximum, when Harmattan winds are the strongest in Sahelo-Sudanian Africa. The similarity in the seasonal patterns of both Harmattan winds and MCM disease cases is obvious, with a strong correlation between the week of winter maximum and that of the onset of epidemic. Similar results, not illustrated here, have been obtained by using surface temperatures and specific humidity for the computation of atmospheric indexes, attesting to the robustness of the analysis.

However, whatever the climatic index is used, this analysis does not allow us to link the intensity of the “epidemic” (the annual number of cases) to the intensity of winter in terms of absolute humidity and surface wind speed. This lack of a relation may be due to the time series length, with an insufficient number of years to study interannual variations, or it may imply that the climatic influence is limited to explaining the occurrence of the seasonal cycle of the epidemic and its geographical range distribution, but not its intensity. Although they fail to forecast epidemic intensity, such climatic indexes, with their correlation with the onset and the seasonal course of the epidemic in Sahel, provide an important means of disease monitoring and prediction in Africa. Indeed, the seasonal pattern of humidity and Harmattan winds can be easily tracked, thus promoting the emergence of an Early Warning Index (EWI) for the onset of MCM epidemics. The seasonal forecast of this EWI based on Harmattan winds could thus be implemented routinely by using comprehensive coupled models of the atmosphere, oceans, and land surface that provide a degree of predictability of climate fluctuations with a seasonal lead time in many parts of the world [22]. The ability of the climate models to predict the winter maximum could be tested by using the outputs of the Development of a European Multi-Model Ensemble System for Seasonal to Interannual Prediction (DEMETER) project, which was conceived and funded under the European Union Fifth Framework Environment Programme. The principal aim of DEMETER was to advance the concept of multimodel ensemble prediction by using a number of state-of-the-art global-coupled ocean–atmosphere models and to produce a series of 6-mo multimodel ensemble hindcasts. The DEMETER project already has application partners in agronomy and in tropical disease prediction [22].

This EWI parameter, in association with other environmental parameters implicated in disease resurgence [23], could help to more precisely characterize disease risk maps at regional scales. The natural extension of this work is to relate this information on the timing of disease outbreaks with specific spatial environmental characteristics at finer scales, in an Early Warning System based on the monitoring of the impact of climate variability and environmental change on epidemic occurrence in West Africa. Recent findings by Molesworth et al. [23] have already quantified the relationship between the environment and the location of the epidemics to propose a model based on environmental variables and to identify regions at risk for meningitis epidemics. The combination of the EWI for MCM epidemic onset and risk maps at regional scales could be a starting point to more optimally direct national and international health policy strategies and to optimize mass vaccination campaigns. In addition, more general measures can be taken by national authorities to improve the control of MCM disease, such as closing markets and schools and discouraging social gatherings when an outbreak is likely to occur [9].

**Patient Summary**

**Background**

Climate is known to be one of the factors that can affect when and how epidemics occur; for example, floods often increase the risk of waterborne disease. However, there are many more subtle climatic changes that might also be important in affecting when and how diseases occur.

**What Did the Researchers Do?**

They looked at the relationship between a recurring epidemic of a disease called meningococcal meningitis in Mali in West Africa and the local climatic conditions, especially the winds. Meningococcal meningitis is a serious infection of the lining of the brain and spinal cord by a bacterium (called Neisseria meningitides). These researchers had previously published some detailed work on the local climate in a French journal. In this paper they have focussed more on the aspects that deal with disease. They found out that over several years the onset of the epidemic coincided with the peak of the winds.

**Who Will Use These Results?**

People who would find these results useful are those who plan for epidemics. Such information will allow them to plan in advance, and even predict whether an epidemic will occur at all. However, these results were based on only the years between 1994 and 2002, and so will need to be confirmed in more years.

## Abbreviations

CSF: cerebrospinal fluid
DEMETER: Development of a European Multi-Model Ensemble System for Seasonal to Interannual Prediction
EWI****: Early Warning Index
MCM: meningococcal meningitis
NCAR: National Center of Atmospheric Research
NCEP: National Centers for Environmental Prediction
PC: principal component
PCA: principal component analysis
WHO: World Health Organization
